# Dietary Polyphenols and Human Health

**DOI:** 10.3390/nu12092893

**Published:** 2020-09-22

**Authors:** Anna Tresserra-Rimbau

**Affiliations:** 1 Department of Nutrition, Food Science and Gastronomy, XaRTA, INSA, School of Pharmacy and Food Sciences, University of Barcelona, 08028 Barcelona, Spain; annatresserra@ub.edu; 2 Centro de Investigación Biomédica en Red Fisiopatología de la Obesidad y la Nutrición (CIBEROBN), Instituto de Salud Carlos III, 28029 Madrid, Spain

Plant-based foods are the main source of phytochemicals, including polyphenols, a large family of compounds with highly diverse chemical structures. The impact of polyphenols, ranging from simple gallic acid to the most complex proanthocyanidins, on different biological processes has been irrefutably demonstrated by numerous studies [1].

Multiple approaches, each with their strengths and weaknesses, have been used to investigate the effects of polyphenols, all making an important and complementary contribution to the field. In vitro and in vivo experimental models play a vital role in the elucidation of the mechanisms of action underlying the health benefits observed in human trials. However, their results cannot always be easily extrapolated to human beings, partly because of considerable interindividual variability and other external factors. For instance, potential effect-modulating variables, such as sex, age, smoking habits, body mass index, and hormone levels, need to be identified, as does the influence of other foods, nutrients and even culinary techniques [1,2]. Additionally, we should not forget the importance of gut microbiota and genetic polymorphisms, which lead to varied circulating metabolites with different biological activities and health impacts [3].

A more recent approach is the use of omics, an integration of disciplines such as metabolomics, genomics, epigenomics, and foodomics based on cutting-edge experimental techniques, including mass spectrometry. The comprehensive ultra-large data sets they generate allow the scientific community to answer new and complex questions [4].

Daily dietary intake of polyphenols is thought to be approximately 1 g, although this estimate is based on subjective food frequency questionnaires, in which participants tend to overestimate the consumption of healthier items. Moreover, despite the availability of useful and comprehensive databases on polyphenol content in food, the concentrations depend on a wide range of factors, including plant variety, ripeness, environmental conditions, cropping systems, cooking, and storage, all of which add to the complexity of calculating intake [5].

In this Special Issue on “Dietary Polyphenols and Human Health”, a series of 10 papers are presented, including three literature reviews [6,7,8] and seven original research papers [9,10,11,12,13,14,15]. The described research contributes to filling some of the gaps in our knowledge about the beneficial effects of dietary polyphenols on chronic health conditions, notably cardiovascular disease, type 2 diabetes, neurological impairment, and also certain risk factors.

In their review, Sandoval et al. describe the molecular mechanisms and signaling pathways involved in the metabolic impact of each group of flavonoids on obesity and related disorders, focusing on the liver, white and brown adipose tissue and central nervous system [6]. Márquez-Campos et al. have collected and summarized the available literature on the antidiabetic effects of both parent flavan-3-ol compounds and their microbial metabolites. The role of microbiota is especially relevant, as flavan-3-ols are poorly absorbed and their metabolization and absorption largely depend on the activity of colonic bacteria [7]. In the third review, Domínguez-López et al. explore the effects of phytoestrogens on human hormone-dependent outcomes throughout the human lifespan, divided into stages of pregnancy, childhood, adulthood, and the pre- and post-menopause [8].

Individual phytoestrogens are also the subject of a cross-sectional study by Sun et al., who are interested specifically in their impact on sleep quality. The association between urinary phytoestrogens (enterolactone, enterodiol, daidzein, O-desmethylangolensin, equol, and genistein) and sleeping disorders and sleep duration was examined in adults from the National Health and Nutrition Examination Survey 2005–2010. Discrepant results were found, depending on the metabolites and the race and sex of the participants, revealing the need for further studies with prospective cohorts and clinical trials [9].

Two of the other papers report clinical trials on the effect of polyphenols on the brain. In a study on psychological well-being (the PPhIT study), Kontogianni et al. concluded that participants with a high polyphenol intake had fewer depressive symptoms and better general mental and physical health compared to those on a low-phenolic diet [10]. The crossover study on mood and cognitive function performed by Wightman et al., where healthy participants received a single dose of a polyphenol-rich extract obtained from mango leaves (<60% mangiferin), revealed no significant results for mood, but cognitive function was enhanced [11].

Taking on the challenge of assessing polyphenol intake, Martini et al. used food frequency questionnaires to compare the nutrients afforded by two different dietary patterns (polyphenol rich and control) in older participants of the MaPLE study. Their ultimate goal is to develop dietary guidelines to increase the intake of these bioactive compounds [12]. Castro-Barquero et al. also used food frequency questionnaires to make a detailed estimation of the polyphenol intake in high cardiovascular risk participants of the PREDIMEDplus study. Monitoring metabolic syndrome symptoms, they found that some phenolic groups were inversely associated with better values of blood pressure, fasting plasma glucose, HDL cholesterol, and triglycerides [13]. Interestingly, both MaPLE and the PREDIMEDplus studies gave similar values for polyphenol intake.

The final two publications shed light on the mechanism of action of polyphenols. Saji et al. explore how a rice bran phenolic extract could target metabolic pathways associated with Type 2 diabetes mellitus, concluding that it modulated the expression of genes involved in β-cell dysfunction and insulin secretion through different mechanisms [14]. Focusing on the pathogenesis of cardiovascular diseases, Nignpense et al. performed a clinical trial with healthy volunteers to evaluate the effect of ingesting a sorghum extract. Although oxidative stress-related endothelial dysfunction and platelet aggregation were not reduced, a beneficial impact on platelet activation and platelet microparticle release was observed [15].

The growth of publications on bioactive compounds in the last years reflects the considerable interest of the scientific community in the field, but a great deal of research still needs to be done. A better understanding of the health benefits of polyphenols and their mechanisms of action will lead to improved (and perhaps individualized) nutritional recommendations aimed at enhancing human health.
